# Melatonin Improved Waterlogging Tolerance in Alfalfa (*Medicago sativa*) by Reprogramming Polyamine and Ethylene Metabolism

**DOI:** 10.3389/fpls.2019.00044

**Published:** 2019-02-01

**Authors:** Qiang Zhang, Xiaofei Liu, Zhifei Zhang, Ningfang Liu, Danzhu Li, Longxing Hu

**Affiliations:** Department of Pratacultural Sciences, College of Agriculture, Hunan Agricultural University, Changsha, China

**Keywords:** alfalfa, ethylene, melatonin, polyamine, waterlogging

## Abstract

Melatonin (MT), polyamines (PAs), and ethylene have been suggested to play key roles in plant growth and development in response to environmental abiotic stresses. However, the effect of melatonin on polyamine and ethylene metabolism under waterlogging stress has rarely been elucidated. The main purpose of this study was to investigate the effect of melatonin pretreatment on waterlogging stress in alfalfa. The experiment was arranged into four treatment groups control with water pretreatment (CK-MT), control with melatonin pretreatment (CK+MT), waterlogging pretreated with water (WL-MT) and waterlogging pretreated with melatonin (WL+MT), with three replications. Six-week-old alfalfa seedlings were pretreated with 100 μM melatonin and exposed to waterlogging stress for 10 days. Plant growth rate, different physiological characteristics, and gene expression level were measured. Results showed that waterlogging induced melatonin accumulation, and melatonin pretreatment increased endogenous MT levels for the control and water-logged plants. Waterlogging stress caused a significant reduction in plant growth, chlorophyll content, photochemical efficiency (Fv/Fm) and net photosynthetic rate (P_n_), while also causing increased leaf electrolyte leakage (EL) and malondialdehyde (MDA) content. Pretreatment with melatonin alleviated the waterlogging-induced damage and reduction in plant growth, chlorophyll content, Fv/Fm and P_n_. Waterlogging stress significantly increased leaf polyamines (Put, Spd, Spm) and ethylene levels, and the increased PAs and ethylene levels are coupled with higher metabolic enzymes and gene expressions. While pretreatment with melatonin further increased Put, Spd and Spm levels, it also decreased ethylene levels under waterlogging, and those increased PAs levels or decreased ethylene levels are regulated by the metabolic enzymes and gene expressions. The results in this study provide more comprehensive insight into the physiological and molecular mechanisms of melatonin-improved waterlogging tolerance in alfalfa. Furthermore, they suggested that melatonin improved waterlogging tolerance in alfalfa at least partially by reprogramming ethylene and PA biosynthesis, attributable to the increased PAs and decreased ethylene levels, which leads to more enhanced membrane stability and photosynthesis as well as less leaf senescence caused by ethylene.

## Introduction

Waterlogging has been suggested as a major environmental stress that affects crop survival, growth, and productivity in those areas prone to heavy rainfall, poor soil drainage as well as high water table fluctuations (Jackson and Colmer, 2005). The availability of molecular oxygen is required to support metabolism and growth of higher plants. However, excess water in the soil often results in inadequate provision of oxygen to the plant cells, causing several phenotypic, physiological and metabolic disturbances, including growth inhibition of shoots and roots, reduction in water and nutrient uptake, leaf photosynthesis and photochemical efficiency as well as root respiratory disturbances and leaf senescence (Boru et al., 2003). In addition, waterlogging often results in a significant increase in ethylene levels in plants. The high ethylene level under waterlogging stress often causes a significant decrease in shoot and root growth, inducing leaf senescence and abscission (Najeeb et al., 2018) as well as a reduction in photosynthesis (Rajala and Peltonen-Sainio, 2001; Pierik et al., 2007). Ethylene biosynthesis begins with the formation of *S*-adenosyl-L-methionine (SAM) from methionine by SAM synthetase. The 1-aminocyclopropane-1-carboxylic acid (ACC) synthase (ACS) then catalyzes the production of ACC from SAM followed by its oxidation to ethylene by ACC oxidase (ACO) (Najeeb et al., 2018).

Polyamines (PAs) are low molecular weight and aliphatic nitrogenous compounds (Groppa and Benavides, 2008), which include spermidine (Spd), spermine (Spm), and putrescine (Put). They have been regarded as a class of plant growth regulators and suggested to be involved in plant growth and development (Tavladoraki et al., 2012). In addition, numerous studies have implied the associations of PA metabolism with plant responses to environmental stress conditions, including drought, high temperature, salinity, nutrient deficiency, and others (Zhao et al., 2017), because of their roles in membrane stability, scavenging free radicals and preserving nucleic acids and proteins structures (Alcázar et al., 2010). PA synthesis and catabolism have been well-illustrated in plants. Ornithine decarboxylase (ODC), ADC, Spd synthase (SPDS), Spm synthase (SPMS), and SAM decarboxylase (SAMDC) are involved in PA synthesis, and DAO and polyamine oxidase (PAO) are involved in the catabolism of PAs in plant tissues (Alcázar et al., 2010). Generally, the biosynthesis and catabolism of PAs are interconnected with other metabolic pathways that function in plant stress tolerance (Alcázar et al., 2010). For example, the interconnection between the stress-induced PA and ethylene metabolism reflect the fact that their biosynthethic pathways share SAM as a substrate, and the functions of PAs and ethylene differ diametrically (Apelbaum et al., 1981).

Melatonin (*N*-acetyl-5-methoxytryptamine) is a low molecular-weight indole amine synthesized from the essential amino acid L-tryptophan (Koyama et al., 2013) and has been reported as a universal signaling molecule in mammals and plant species (Hardeland et al., 2011; Tan et al., 2012). Numerous studies have implied the essential role of melatonin in plant growth and development and in protecting plants from environmental stressors, including heat and cold temperatures, water stress, osmotic and ionic stress, ultraviolet radiation and heavy metal stress (Tan et al., 2012; Shi and Chan, 2014; Shi et al., 2015; Gong et al., 2017; Zhao et al., 2017).

Alfalfa (*Medicago sativa*) is an important perennial forage grass worldwide because of its high yield and high quality. Introduction of alfalfa into south China has the potential for alleviating shortages of good forage that limit the development of herbivorous animal husbandry. However, the growth range of alfalfa is limited due to its sensitivity to waterlogging or flooding in Southern China (Smethurst et al., 2005).

It has been suggested that melatonin can regulate PA levels in rat brains and human skin (Lee et al., 2000, 2003). However, few studies have been conducted to elucidate how melatonin modulates PA metabolism in response to abiotic stress in plants, such as waterlogging. Melatonin may exert its protective effects through PAs and ethylene metabolism under waterlogging stress in plants. In this study, the effects of melatonin pretreatment on polyamine and ethylene metabolism were investigated in alfalfa leaves under waterlogging stress. Polyamines and ethylene levels, key metabolic enzymatic activities and their respective gene expression levels were determined after 10 days of waterlogging stress was imposed.

## Materials and Methods

### Plant Materials and Growth Conditions

Alfalfa ‘55v48’ cultivars, supplied by Beijing RYTWAY, were used in our study. Twenty-five seeds were planted in each pot (10 cm × 10 cm in diameter and height) with 16 pots in total, which were filled with a mixture of loamy topsoil and sand (1:2, v/v). Seedlings were cultured in the greenhouse with the following environmental conditions: 18/25°C temperature (day/night), 14 h/10 h photoperiod, 500–550 μmol m^-2^ s^-1^ light intensity, and 60–85% relative humidity. The plants were fertilized weekly with Hoagland’s liquid solution.

### Treatments and Experimental Design

Plants at six- to seven-leaf stages (about 6 weeks after sowing) were exposed to soil waterlogging. A preliminary study showed that 100 μM melatonin was the most effective concentration to improve waterlogging tolerance. At 1 day prior to waterlogging, 100 μM of melatonin were foliar sprayed for 15 ml per pot and the non-melatonin treatment were pretreated with water. Plants were subjected to waterlogging by immersing the plastic pots into water-filled plastic tubs by maintaining 1 cm water layer above the soil surface and last for 10 days, whereas the control pots were watered regularly to field capacity. There are four treatments in this experiment: control with water pretreatment (CK-MT), control with melatonin pretreatment (CK+MT), waterlogging pretreated with water (WL-MT) and waterlogging pretreated with melatonin (WL+MT). All pots with four treatments were divided into three groups and maintained in three growth chambers (three replicates), with a temperature of 20/25°C (day/night), 14 h/10 h photoperiod, 60–85% relative humidity and a light intensity of 500 μmol m^-2^ s^-1^. Plants were sampled at 10 days of waterlogging stress for physiological and PA analysis and harvested at 0, 4, 24 h and 5, 10 days after waterlogging stress was imposed for molecular analysis.

### Determination of Plant Growth Rate

Plant growth rate was determined by measuring the shoot height with three seedlings per pot and calculating the difference before and after waterlogging stress was imposed.

### Determination of Leaf Chlorophyll Content

Leaf chlorophyll content was measured on the fourth leaves from the top by using a hand-held chlorophyll meter (SPAD-502, Minolta, Corp., Spectrum Technologies).

### Determination of Leaf Photochemical Efficiency

Leaf photochemical efficiency (Fv/Fm) was evaluated by using a chlorophyll fluorometer (OS1-FL, Opti-Sciences, Hudson, NH, United States). Plants were adapted in darkness for 30 min and the then the measurements were made on intact leaves with the fluorometer.

### Determination of Leaf Net Photosynthetic Rate (P_n_)

Net photosynthetic rate (P_n_) was measured in the third leaves by using a gas analyzer (Li-6400, LICOR, Inc., Lincoln, NE, United States) with controlled conditions (400 μmol mol^-1^ CO_2_, 500 μmol s^-1^ flow rate) and a Licor 6400 LED external light source providing a photosynthetic photon flux density of 500 μmol m^-2^ s^-1^. Net photosynthetic rate was measured for three subsamples in each pot.

### Determination of Leaf Electrolyte Leakage (EL)

Leaf electrolyte leakage (EL) was measured using ≈0.1 g fresh samples. Leaf segments were immersed in a 50 ml tube filled with 15 ml of deionized water and then shaken for 24 h at room temperature. The initial conductance (Ci) was recorded using a conductance meter (YSI-3100; Guangzhou, China). Leaf segments in the 50 ml tube were then autoclaved at 120°C for 30 min. The maximum conductance (Cmax) of the incubation solution with killed tissues was recorded after the solution cooled to room temperature. Relative EL was calculated as (Ci/Cmax) × 100.

### Determination of Leaf Malondialdehyde (MDA)

For leaf malondialdehyde (MDA) measurement, leaf samples were homogenized with ice-cold extraction buffer (100 mM PBS, pH 8.0). After that, the extraction was then centrifuged at 14,000 *g* for 20 min at 4°C. The supernatant was transferred to a new tube and used for the determination of MDA content according to the method of Heath and Packer (1968).

### Determination of Leaf Endogenous Melatonin Levels

Endogenous melatonin levels of alfalfa leaves were determined according to the method described by Shi and Chan (2014). Briefly, 1 g of alfalfa tissues was homogenized thoroughly in 5 ml of extraction solution (acetone: methanol: water = 89: 10: 1). The supernatant was transferred to a new tube containing 0.5 ml of 1% trichloric acid for protein precipitation after being centrifuged at 1000 *g* for 10 min at 4°C. Then, the centrifuged extract was used for quantification of melatonin using the Melatonin ELISA Kit (EK-DSM; Buhlmann Laboratories AG, Schönenbuch, Switzerland) according to the user manuals.

### Determination of Leaf Free PA Levels

Leaf free PAs were extracted as the approaches described by Duan et al. (2008) with modifications. Briefly, 0.4 g fresh tissues were homogenized in 4 ml of 5% (v/v) cold perchloric acid and incubated at 4°C for 1 h. 1,6-hexanediamine was added as an internal standard prior to centrifugation at 12,000 ×*g* for 30 min at 4°C. An aliquot of the supernatant was reacted with 2 ml of 2N NaOH and 15 μl of benzoyl chloride and then vortexed and incubated for 30 min at 37°C, and then 4 ml saturated NaCl was added to terminate the reaction. The diethyl ether extracted benzoyl PAs were evaporated to dryness and re-dissolved in 1 ml methanol. The benzoyl derivatives were separated and analyzed by an HPLC system (Waters Series HPLC, Milford, MA, United States). Samples were injected with a volume of 20 μl into a 20-μl loop on a C18 reverse-phase column (250 mm × 2.1 mm, 5 μm; Supelco Analytical, Bellefonte, PA, United States) at room temperature. The samples were then eluted from the C18 column in a gradient program at a flow rate of 0.2 ml/min and detected at 254 nm with a UV detector. The three PA standards (Aladdin, Co.) of Put, Spd, and Spm were prepared at various concentrations for the production of the appropriate standard curves.

### Determination of PA Metabolism Key Enzyme Activities

The activities of ADC, ODC, and SAMDC were tested according to the approaches described by Zhao et al. (1996) and Hu et al. (2012). Briefly, Fresh tissues were homogenized in 100 mM PBS (pH 8.0) containing 0.1 mM PMSF, 1 mM PLP, 5 mM DTT, 5 mM EDTA, 25 mM ASA and 0.1% PVP. The supernatant was then dialyzed after centrifugation at 12,000 ×*g* for 40 min at 4°C. The dialysis was started with the addition of 3 ml 100 mM PBS (pH 8.0) containing 0.05 mM PLP, 1 mM DTT, and 0.1 mM EDTA for 24 h in darkness at 4°C. The dialyzed samples were then used for enzyme determination. 0.3 ml of the dialyzed enzyme extract was mixed with 1 ml of the reaction mixtures containing 100 mM Tris-HCl buffer (pH 7.5), 5 mM EDTA, 50 mM pyridoxal phosphate and 5 mM DTT. After which, 0.2 ml of 25 mM L-arginine, L-ornithine, or *S*-adenosyl methionine was added respectively. The mixtures were incubated at 37°C for 1 h. After that, PCA was added until the final concentration of PCA was 5%. After centrifuging at 3000 ×*g* for 10 min, 0.5 ml of the supernatant was mixed with 1 ml of 2 mM NaOH and 10 μl benzoyl chloride and then stirred for 20 s. After incubation for another 30 min at 37°C, 4 ml saturated NaCl solution and 3 ml ether were added to the mixture. 2 ml of the ether phase was evaporated to dryness and re-dissolved in 1 ml 60% methyl alcohol. The absorbance of this solution was measured at 254 nm using a Shimadzu UV1800 spectrophotometer (Shimadzu, Co., Ltd., Japan). Enzyme activities were expressed in nmol Arg g^-1^ FW h^-1^, nmol Put g^-1^ FW h^-1^, and nmol SAM g^-1^ FW h^-1^.

The activities of DAO and PAO were measured according to the method described by Hu et al. (2012). Briefly, 0.4 g fresh leaves were homogenized using pre-chilled mortar and pestle at 4°C in 100 mM sodium phosphate buffer (pH 6.5). The homogenates were then centrifuged at 10,000 *g* for 20 min at 4°C and the supernatants were used for the determination of DAO and PAO activities. The reactions were started by adding 15 μl of Put (for DAO measurement) and 15 μl of Spd + Spm (for PAO measurement) to the mixture of the supernatant with 4-aminoantipyrine/*N*, *N*-dimethylaniline reaction solution and 0.1 ml horseradish peroxidase (250 U ml^-1^). A unit of enzyme activity was defined as one giving a change in optical density of 0.001 absorbance units at 254 nm (Zhao et al., 2017).

### Determination of Leaf Ethylene Production Rate and Key Enzyme Gene Expressions

The ethylene production rate was assayed as the method described by Wilkinson and Davies (2009) with modification. Briefly, 0.5 g of the third expanded leaves (from top) were collected at 0, 4, 24 h and 5, 10 days of waterlogging stress, and put into 25 ml sealed glass vials. Samples were held for 5 h at 25°C under illumination with 5000 Lx. Gas samples (1 ml) were withdrawn from the vial head space with a disposable plastic syringe and manually injected into a gas chromatograph (5890 C, Agilent Technologies UK, Ltd., Wokingham, United Kingdom). The temperature was maintained at 100°C for 5 min to resolve ethylene, increased at 15°C min^-1^ to 150°C and held for 1.5 min to remove any water vapor introduced into the column in the sample injection. The helium carrier gas was set at a flow rate of 5.7 ml min^-1^ and detection was performed by flame ionization. Ethylene concentration was calculated with reference to peak areas of known ethylene standards (BOC Special Gases, Manchester, United Kingdom) and the ethylene emission rate was calculated for tissue fresh weight and the duration of incubation (Wilkinson and Davies, 2009). The amount of released ethylene was expressed in μl per g plant tissue per hour. The measurement was conducted in three biological replicates.

Total RNA extraction, cDNA synthesis, and qRT-PCRs were performed as Hu et al. (2016) described. Briefly, 0.1 g fresh tissues were used for total RNA extraction by using Trizol reagent (Invitrogen, Carlsbad, CA, United States). RNA quality and integrity were checked by Nanodrop 2000 and 0.8% agarose gel. Then, the first strand cDNA were synthesized from 2 μg of total RNA using oligo(dT)12-18 primer with the cDNA synthesis kit (Fermentas, Burlington, ON, Canada). Gene-specific primers were designed based on the target gene sequences using Primer 5 software (Table 1). The real-time RT-PCR was conducted in ABI7500 with a final volume of 20 μl. The real-time PCR analysis contained three independent biological replicates and two technical replicates for each sample. The PCR conditions were as follows: 40 cycles of 95°C denaturation for 5 s, and 52∼55°C annealing and extension for 20 s. The relative expression level of genes for each sample was calculated relative to a calibrator using the DDCT method as described by Livak and Schmittgen (2001).

**Table 1:** Details of primers used for analyzing the expression of genes involved in the biosynthesis of polyamines in alfalfa by quantitative real-time PCR.

Gene	Primer	Nucleotide sequence	Accession
*SAMDC*	SAMDC-F SAMDC-R	5′ -CAA CGG TGG CGT AGA AAA AT-3′ 5′-GCC TTC AAA ACC GAT AGC TG-3′	CB891404^∗^
*SPDS*	SPDS-F SPDS-R	5′-AAG GGA TGA GTG TGC GTA CC-3′ 5′-TTT GGA GAC ATC GAC AAC CA-3′	TA22135_3880^∗^
*SPMS*	SPMS-F SPMS-R	5′-GCC AGT GAA GAA AAG GGT CA-3′ 5′-AGA ACC ACC CAG AAA CAA CG-3′	TA27030_3880^∗^
*ADC*	ADC-F ADC-R	5′-CTG GCC ATT TTG GTT CAA CT-3′ 5′-ACC CAA ACG AAG CAA TTC AC-3′	TA20893_3880^∗^
*PAO*	PAO-F PAO-R	5′-TTT TGG CAG CAC ATG GAT AA-3′ 5′-TTA TTC CAC CAG CAG GGA AC-3′	BQ122766^∗^
*DAO*	DAO-F DAO-R	5′-TGC AAT CCC AGA TGA AGT GA-3′ 5′-CAG CTA GCA ATG TGC CAT GT-3′	AJ500329^∗^
*MtACS3*	ACS-F ACS-R	5′-GTCTACCAGGTTTCAGAGTTG-3′ 5′-CTCTTCTTCAATCTTTCCCTAT-3′	MTR 8g101820^##^
*MtACO1*	ACO-F ACO-R	5′-CCAAAGGGCTAGAGGCTGTTC-3′ 5′-GGTAGGTGACGCAAATGGAAA-3′	MTR3g083370^##^
*MsETR8*	ETR -F ETR -R	5′-GTGACAACATCTCTGACCCGT-3′ 5′-ACCCTGCTTCCTTCCCTTGAT-3′	JF965422^##^
*MtActin*	Actin -F Actin -R	5′-ACGAGCGTTTCA GATG-3′ 5′-ACCTCCGATCCAGACA-3′	MTR 7g026230^##^


*^##∗^Medicago EST sequence Accession no*.

### Statistical Analysis

Data statistical analysis was conducted following the ANOVA analysis of variance using SAS for Windows (SAS Institute, Cary, NC, United States). Means and standard errors were calculated for three replicates. Comparisons between means were carried out using Duncan’s multiple range tests at a significance level of *p* < 0.05.

## Results

### Exogenous Melatonin Alleviates Waterlogging-Induced Growth Inhibition in Alfalfa Plants

**FIGURE 1:**
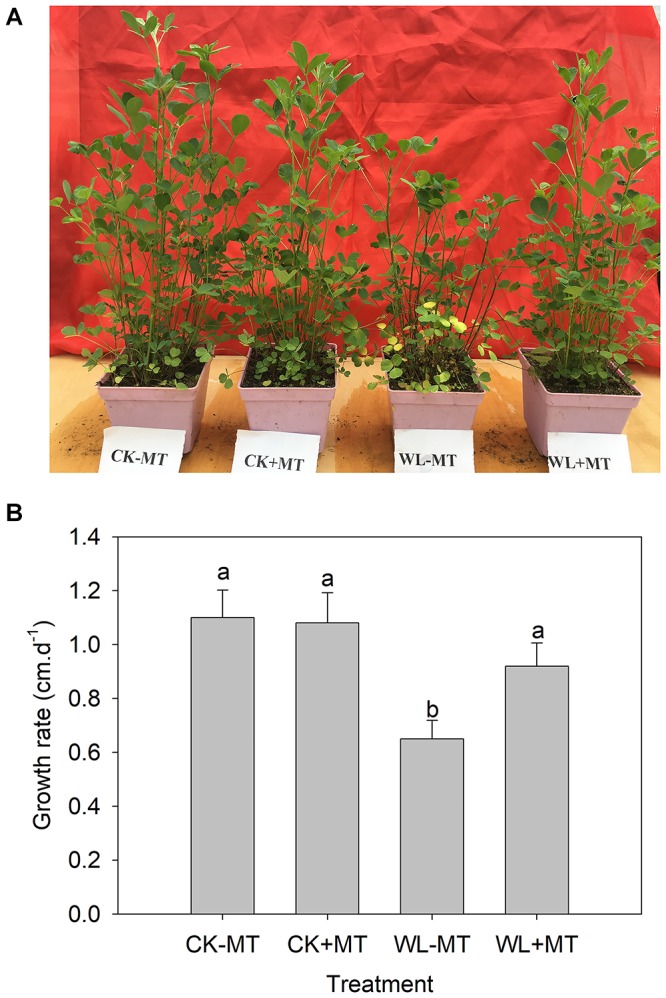
Effect of melatonin (MT) pretreatment on alfalfa plants under control and waterlogging stress. The representative picture **(A)** and shoot growth rate **(B)** of 45-day-old plants of the alfalfa cultivars ‘55v48’ pretreated with 100 μM MT and then subjected to waterlogging stress for 10 days. Vertical bars on the top indicate the means ± SD (*n* = 4). Letters marked by the same letters are not significant at *P* < 0.05 (Ducan’s multiple range test).

Prolonged waterlogging stress (10 days) caused significant growth inhibition and leaf necrosis as demonstrated by Figure 1A,B. The growth rate of the alfalfa plants decreased from about 1.1 cm per day under control conditions to about 0.65 cm per day under waterlogging stress (Figure 1A,B). However, a remarkable alleviating effect was observed in melatonin pretreated plants subjected to waterlogging stress for 10 days. The plants pretreated with melatonin grown in control conditions displayed similar phenotypes with the control plants and showed no signs of damage symptoms.

### Waterlogging Stress and Exogenous Melatonin Increased Endogenous Melatonin Content in Alfalfa

**FIGURE 2:**
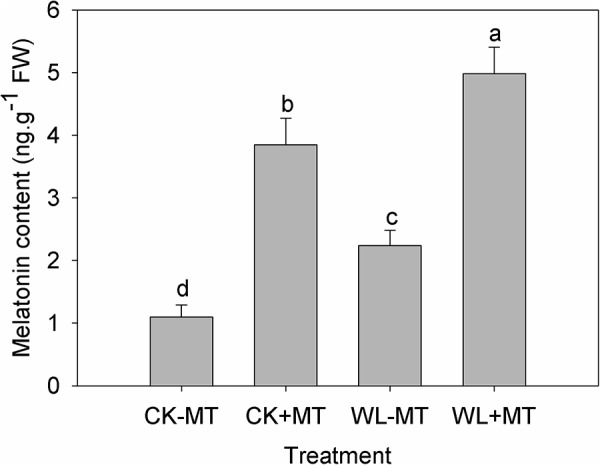
Effects of waterlogging (WL) and exogenous melatonin (MT) pretreatment on the endogenous melatonin content in the leaves of alfalfa. 45-day-old alfalfa plants pretreated with 100 μM melatonin and then exposed to 10 days of waterlogging stress. CK-MT, control without melatonin pretreatment; CK+MT, control with melatonin pretreatment; WL-MT, waterlogging without melatonin pretreatment; WL+MT, waterlogging with melatonin pretreatment. Data are expressed as mean ± SE of four replications. Letters marked by the same letters are not significant at *P* < 0.05 (Ducan’s multiple range test).

To determine whether the waterlogging stress and exogenous melatonin influence the endogenous melatonin levels, the endogenous melatonin concentration of alfalfa leaves was quantified after 10 days of waterlogging treatment. The melatonin concentration was about 1.1 ng g^-1^ fresh weight for the plants grown at control conditions (CK-MT) (Figure 2). Waterlogging (WL-MT) stress substantially increased the endogenous melatonin content in leaves of alfalfa, which increased 2.0-fold when compared to the control levels (CK-MT). However, pretreatment with exogenous melatonin resulted in a significant increase in melatonin content for the control plants (CK+MT) and water-logged plants (WL+MT), which enhanced 3.5- and 4.5-fold as compared to the control plants (CK-MT).

### Exogenous Melatonin Improved Waterlogging Stress-Induced Membrane Damage

**FIGURE 3:**
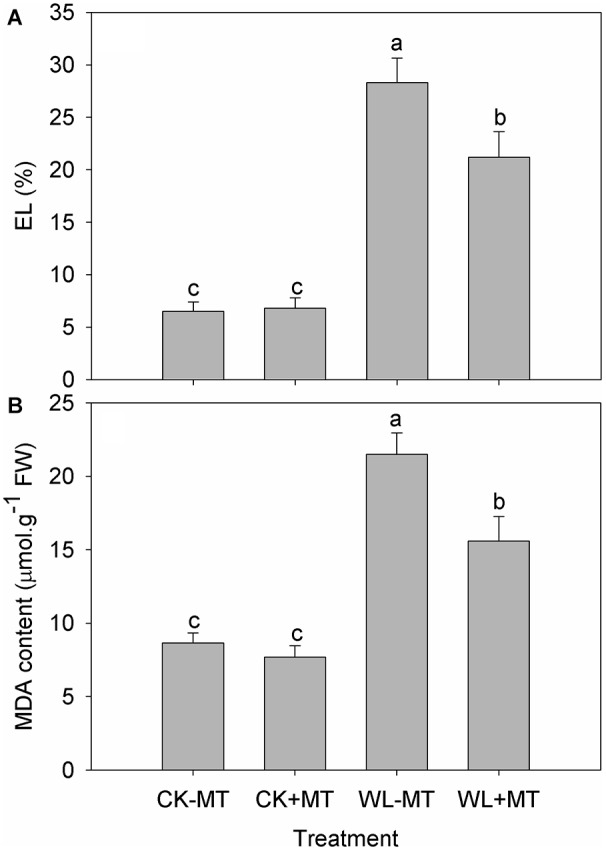
Effects of waterlogging (WL) and exogenous melatonin (MT) pretreatment on the leaf electrolyte leakage (EL, **A**) and MDA **(B)** content in alfalfa. 45-day-old alfalfa plants pretreated with 100 μM melatonin and then exposed to 10 days of waterlogging stress. CK-MT, control without melatonin pretreatment; CK+MT, control with melatonin pretreatment; WL-MT, waterlogging without melatonin pretreatment; WL+MT, waterlogging with melatonin pretreatment. Data are expressed as mean ± SE of four replications. Letters marked by the same letters are not significant at *P* < 0.05 (Ducan’s multiple range test).

Waterlogging stress caused a significant increase in leaf EL (Figure 3A) and malonaldehyde (MDA) content (Figure 3B), which increased to 4.4- and 2.5-fold of the control level for EL and MDA content after 10 days of exposure, respectively. Instead, the leaf EL and MDA content significantly reduced in the waterlogging-stressed plants after melatonin pretreatment, which decreased by 25% for EL and 27% for MDA after 10 days of exposure when compared to the waterlogging stress alone (Figure 3A,B). Pretreatment with melatonin had no effect on leaf EL and MDA content in alfalfa under control conditions.

### Effect of Exogenous Melatonin Improved Waterlogging Stress-Induced Leaf Senescence and Photosynthesis Reduction

**FIGURE 4:**
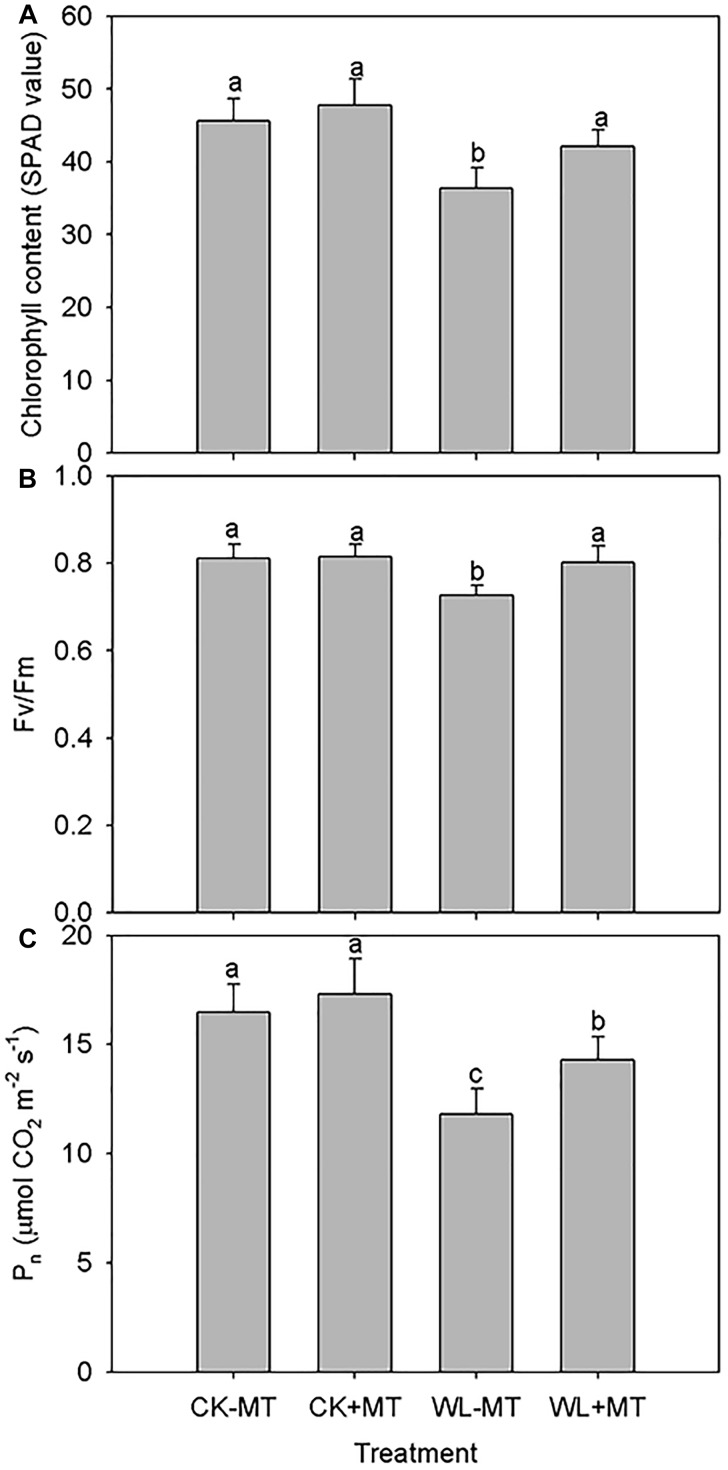
Effects of waterlogging (WL) and exogenous melatonin (MT) pretreatment on the leaf chlorophyll content **(A)**, leaf maximum photochemical efficiency (Fv/Fm, **B**) and net photosynthetic rate (P_n_, **C**) in alfalfa. 45-day-old alfalfa plants pretreated with 100 μM melatonin and then exposed to 10 days of waterlogging stress. CK-MT, control without melatonin pretreatment; CK+MT, control with melatonin pretreatment; WL-MT, waterlogging without melatonin pretreatment; WL+MT, waterlogging with melatonin pretreatment. Data are expressed as mean ± SE of four replications. Letters marked by the same letters are not significant at *P* < 0.05 (Ducan’s multiple range test).

Waterlogging stress caused significant leaf necrosis and senescence as indicated by the reduction of leaf chlorophyll content. Leaf chlorophyll content in water-logged plants was 80% of the water pretreated control level, while chlorophyll content in plants pretreated with melatonin was similar to the water pretreated control level (CK-MT) for both under waterlogged and non-waterlogged conditions (Figure 4A).

The leaf maximum photochemical efficiency (Fv/Fm) and net photosynthetic rate (P_n_) under waterlogging were 11 and 28% lower than in the nation water-logged controls, respectively (Figure 4B,C). Pretreatment with melatonin improved Fv/Fm and P_n_ by 10 and 18% under waterlogging when compared with non-melatonin pretreatment. Melatonin pretreatment had no effect on the Fv/Fm and P_n_ under non-water-logged conditions as compared to the water pretreated plants.

### Exogenous Melatonin Application Improved Free Polyamine Levels

**FIGURE 5:**
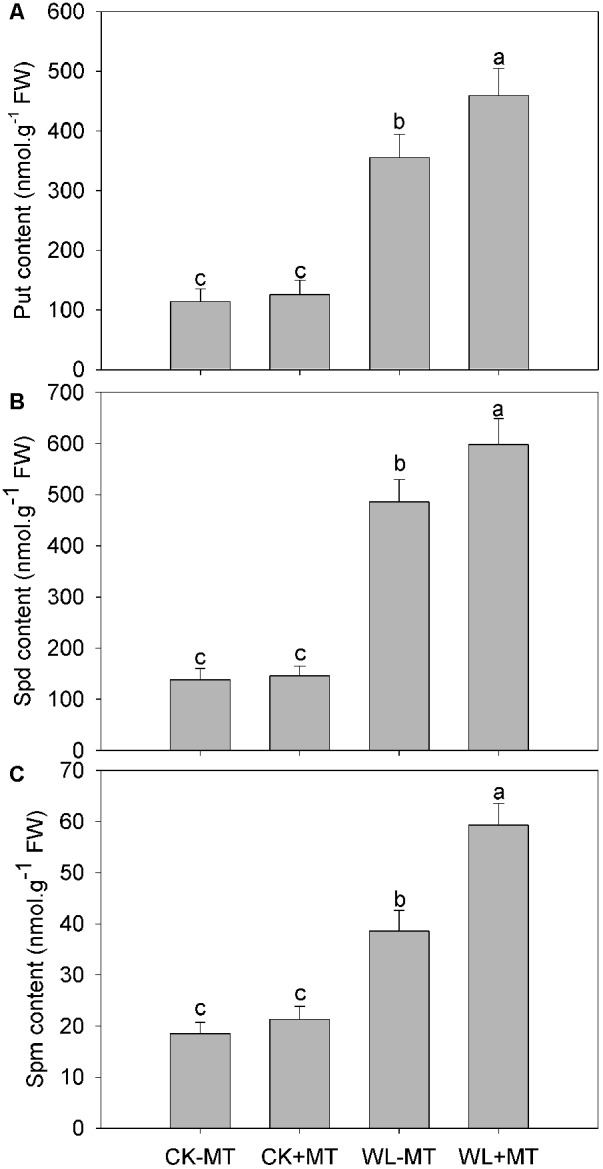
Effects of waterlogging (WL) and exogenous melatonin (MT) pretreatment on leaf putrescine (Put, **A**), spermidine (Spd, **B**) and spermine (Spm, **C**) content in alfalfa plants. 45-day-old alfalfa plants pretreated with 100 μM melatonin and then exposed to 10 days of waterlogging stress. CK-MT, control without melatonin pretreatment; CK+MT, control with melatonin pretreatment; WL-MT, waterlogging without melatonin pretreatment; WL+MT, waterlogging with melatonin pretreatment. Data are expressed as mean ± SE of four replications. Letters marked by the same letters are not significant at *P* < 0.05 (Ducan’s multiple range test).

The levels of the three soluble Pas—Put, Spd, and Spm—ncreased substantially under waterlogging, which increased to 3.1-, 3.5-, and 2.1-fold higher than the control levels, respectively (Figure 5A–C). However, pretreatment with melatonin further increased leaf Put, Spd, and Spm levels under waterlogging, whereas pretreatment with melatonin has no effect on Put, Spd, and Spm levels under control conditions.

### Exogenous Melatonin Application Regulated Key Enzymes Involved in Polyamine Metabolism

**FIGURE 6:**
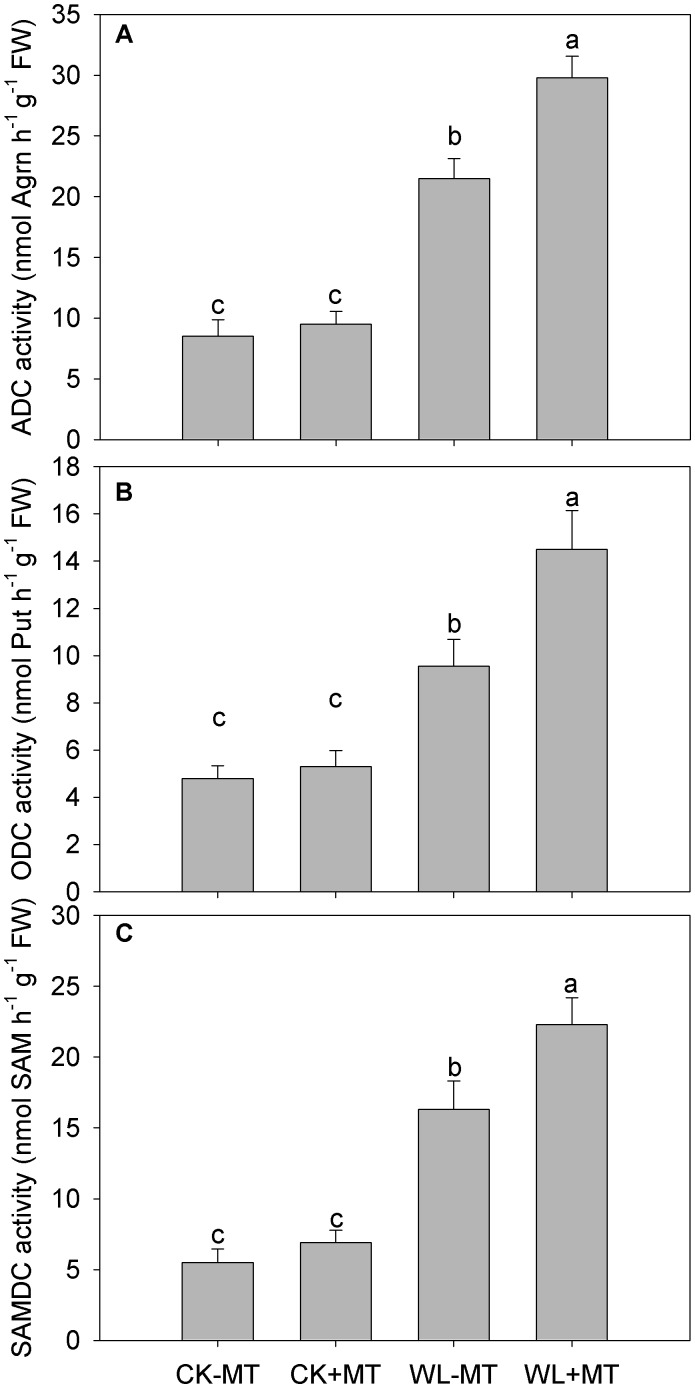
Effect of waterlogging (WL) and exogenous melatonin (MT) pretreatment on ADC **(A)**, ornithine decarboxylase (ODC, **B**) and *S*-adenosylmethionine decarboxylase (SAMDC, **C**) activity in leaves of alfalfa plants. 45-day-old alfalfa plants pretreated with 100 μM melatonin and then exposed to 10 days of waterlogging stress. CK-MT, control without melatonin pretreatment; CK+MT, control with melatonin pretreatment; WL-MT, waterlogging without melatonin pretreatment; WL+MT, waterlogging with melatonin pretreatment. Data are expressed as mean ± SE of four replications. Letters marked by the same letters are not significant at *P* < 0.05 (Ducan’s multiple range test).

Waterlogging stress dramatically increased ADC, ODC, and SAMDC activities in leaves of alfalfa, which increased to 2.5-, 2.0-, and 3.0-fold of the control levels, respectively (Figure 6A–C). Pretreatment with melatonin accelerated the increase and significantly increased ADC, ODC, and SAMDC activities in water-logged plants, while plants pretreated with melatonin had no obvious effect on the ADC, ODC, and SAMDC activities under non-water-logged conditions.

**FIGURE 7:**
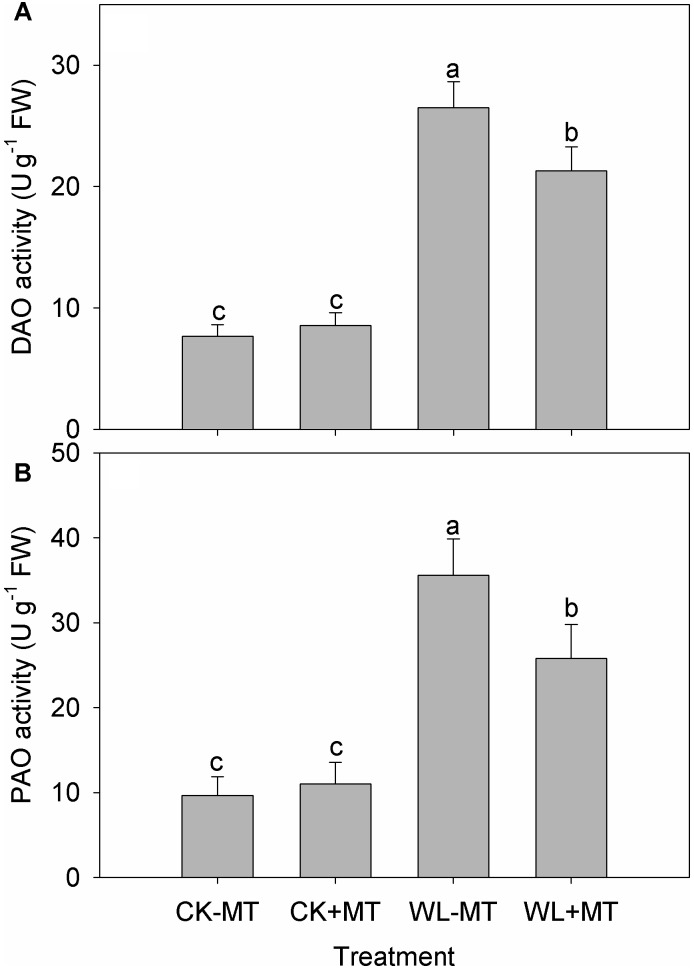
Effects of waterlogging (WL) and exogenous melatonin (MT) pretreatment on leaf DAO **(A)** polyamine oxidase (PAO, **B**) activity in alfalfa plants. 45-day-old alfalfa plants pretreated with 100 μM melatonin and then exposed to 10 days of waterlogging stress. CK-MT, control without melatonin pretreatment; CK+MT, control with melatonin pretreatment; WL-MT, waterlogging without melatonin pretreatment; WL+MT, waterlogging with melatonin pretreatment. Data are expressed as mean ± SE of four replications. Letters marked by the same letters are not significant at *P* < 0.05 (Ducan’s multiple range test).

Waterlogging increased leaf DAO and PAO activity in alfalfa plants, which increased 3.5- and 3.7-fold when compared to the control levels (Figure 7A,B), respectively. However, the DAO and PAO activity was reduced by 24 and 38% in the melatonin pretreated plants under waterlogged conditions as compared to the waterlogged alone.

### Exogenous Melatonin Application Regulated the Expression of Key Genes Involved in Polyamine Metabolism

**FIGURE 8:**
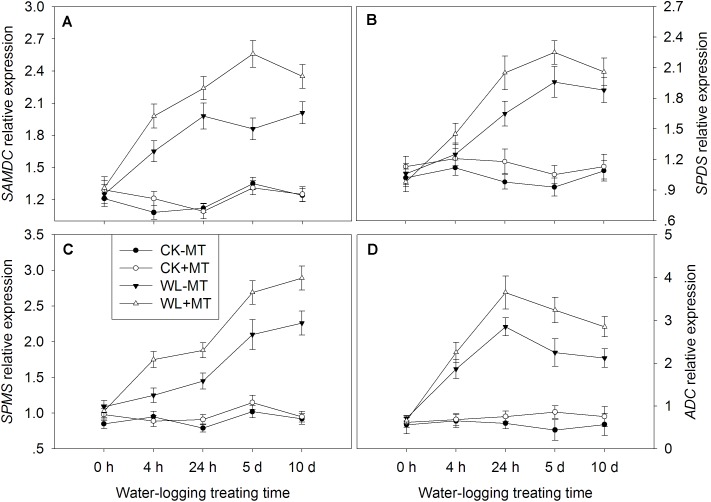
Effects of waterlogging (WL) and exogenous melatonin (MT) pretreatment on SAM decarboxylase (SAMDC, **A**), spermidine synthase (SPDS, **B**), spermine synthase (SPMS, **C**) and ADC **(D)** gene expression in alfalfa plants. 45-day-old alfalfa plants pretreated with 100 μM melatonin and then exposed to 10 days of waterlogging stress. CK-MT, control without melatonin pretreatment; CK+MT, control with melatonin pretreatment; WL-MT, waterlogging without melatonin pretreatment; WL+MT, waterlogging with melatonin pretreatment. Data are expressed as mean ± SE of four replications.

**FIGURE 9:**
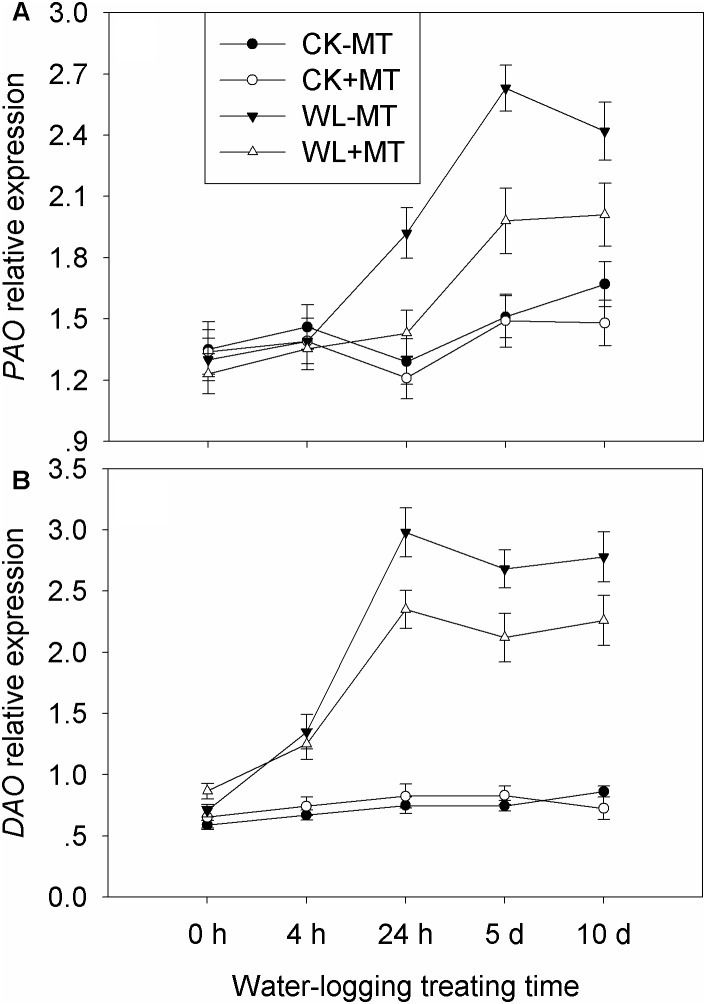
Effects of waterlogging (WL) and exogenous melatonin (MT) pretreatment on DAO **(A)** polyamine oxidase (PAO, **B**) gene expression in alfalfa plants. 45-day-old alfalfa plants pretreated with 100 μM melatonin and then exposed to 10 days of waterlogging stress. CK-MT, control without melatonin pretreatment; CK+MT, control with melatonin pretreatment; WL-MT, waterlogging without melatonin pretreatment; WL+MT, waterlogging with melatonin pretreatment. Data are expressed as mean ± SE of four replications.

The key enzyme genes involved in the polyamine biosynthetic and catabolic pathways, i.e., *SAMDC, SPDS, SPMS*, *ADC*, *DAO*, and *PAO* were analyzed by qRT-PCR (Figure 8). The expression levels of *SAMDC* start to rise 4 h after waterlogging and generally remained high till day 10 (Figure 9A). The expression level of *SAMDC* in waterlogging stress was upregulated by 1.5-fold at 4 h, 1.8-fold at 24 h, 1.4-fold at day 5, and 1.6-fold at day 10 as compared with the control levels. Pretreatment with melatonin caused further upregulation of *SAMDC* during waterlogging treatment, which upregulated 1.8-fold at 4 h, 2.0-fold at 24 h, and 1.9-fold at days 5 and 10 when compared to the control levels. The expression levels of *SPDS* started to rise 24 h after waterlogging and peaked at 5 days (Figure 9B). The expression level of *SPDS* in waterlogging stress was upregulated by 1.7-fold at 24 h, 2.1-fold at day 5, and 1.7-fold at day 10 as compared with the control levels. Pretreatment with melatonin caused further upregulation of *SPDS* during waterlogging treatment, which upregulated 1.3-fold at 4 h, 2.1-fold at 24 h, 2.4-fold at day 5, and 1.9-fold at day 10 when compared to the control levels. Waterlogging induced significant upregulation of *SPMS* in leaves of alfalfa, which upregulated by 1.3-fold at 4 h, 1.8-fold at 24 h, 2.1-fold at day 5, and 2.5-fold at day 10 when compared to the control levels (Figure 8C). However, those upregulated folds rose to 1.8-fold at 4 h, 2.4-fold at 24 h, 2.6-fold at day 5, and 3.1-fold at day 10 by melatonin pretreatment, respectively. The expression level of *ADC* significantly increased with the progress of waterlogging and peaked at 24 h, when compared to the control level (Figure 8D). Melatonin pretreatment substantially upregulated the expression level of *ADC* under waterlogging treatment.

Pretreatment with melatonin alone had no significant effect on the expression levels of *PAO* and *DAO* under control condition (Figure 9). Waterlogging did not significantly affect the expression level of *PAO* during the first 4 h but began to rise 24 h after waterlogging, and the expression levels peaked at 5 days of waterlogging (Figure 9A). Melatonin pretreatment dramatically downregulated the expression levels of *PAO* from 24 h to 10 days when compared with waterlogging alone. Waterlogging stress induced a higher expression level of *DAO* as compared to the control plants (Figure 9B). Evidently, foliar pretreatment with melatonin remarkably depressed the expression levels of *DAO* as compared to waterlogging alone.

### Exogenous Melatonin Application Downregulates Ethylene Synthesis and Signaling

**FIGURE 10:**
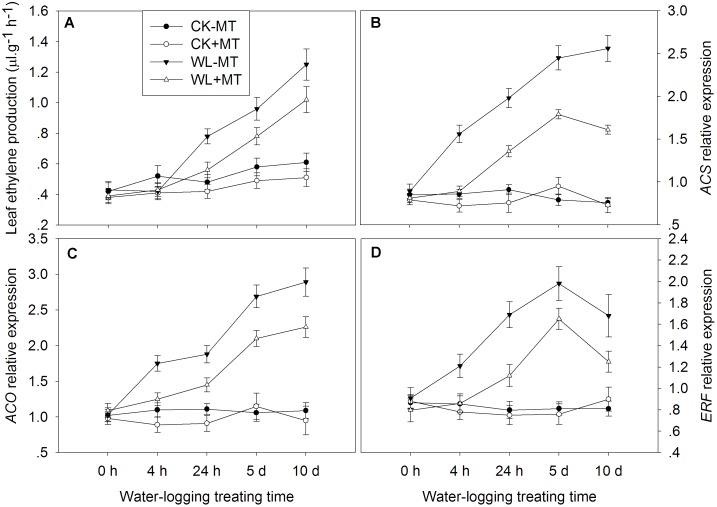
Effect of waterlogging (WL) and exogenous melatonin (MT) pretreatment on ethylene production **(A)**, and key genes involved in ethylene synthesis **(B,C)** and signaling **(D)** in leaves of alfalfa plants. 45-day-old alfalfa plants pretreated with 100 μM melatonin and then exposed to 10 days of waterlogging stress. CK-MT, control without melatonin pretreatment; CK+MT, control with melatonin pretreatment; WL-MT, waterlogging without melatonin pretreatment; WL+MT, waterlogging with melatonin pretreatment. Data are expressed as mean ± SE of four replications.

The leaf ethylene production rate started to rise 4 h after waterlogging and continually increased over the l0 days of treatment (Figure 10A). The amount of ethylene increased to 1.6-, 1.7-, and 2.0-fold of the control levels at 24 h, 5 and 10 days under waterlogging, respectively. However, pretreatment with melatonin dramatically repressed the leaf ethylene production rate under waterlogging. Under water-logged conditions, the leaf ethylene production rate in melatonin pretreated plants decreased by 39, 23, and 22% when compared to the waterlogging alone at 24 h, 5 and 10 days of treatment, respectively.

The genes possibly involved in ethylene biosynthesis (*ACS, ACO*) and signaling (*ERF*) were determined in alfalfa plants. Plants pretreated with melatonin have no obvious effect on the *ACS, ACO*, and *ERF* expression under non-water-logged conditions (Figure 10). Waterlogging dramatically increased the expression levels of *ACS* in leaves of alfalfa, which increased to 1.8-, 2.2-, 3.1-, and 3.4-fold of the control levels at 4 h, 24 h, 5 and 10 days, respectively (Figure 10B). However, pretreatment with melatonin significantly decreased *ACS* expression in water-logged plants as compared to the waterlogging alone. Waterlogging induced a significantly higher expression level of *ACO* and *ERF* over the 10 days of treatment when compared to the control plants (Figure 10C,D). Pretreatment with melatonin remarkably decreased the expression level of *ACO* and *ERF* over the 10 days of treatment when compared to the waterlogging alone.

## Discussion

The excessive water content in soil (waterlogging) is a major environmental stress that limits crop production and causes yield losses worldwide (Jackson and Colmer, 2005). Crops can endure soil waterlogging from some hours to some days or months, depending on the tolerance to flooding for the crop species or cultivars (Voesenek and Bailey-Serres, 2015). However, excess water in soil often exerts detrimental effects to these crops which are intolerant to waterlogging stress. Alfalfa is the most widely used forage legume, but it is susceptible to waterlogging stress, and this is a serious constraint in areas with shallow water tables and heavy rainfalls (Striker and Colmer, 2016).

Waterlogging induces several phenotypic and physiological disturbances, including growth inhibition of roots and shoots, impairs water and nutrient uptake, and eventually results in plants chlorosis and even death (Arbona et al., 2008). Here, waterlogging caused a dramatic increase in EL and MDA content and a remarkable decrease in chlorophyll content in alfalfa plants, but this response was greatly suppressed by melatonin pretreatment. These results implied that melatonin pretreatment can alleviate the detrimental effect of waterlogging in alfalfa. To confirm this finding, we also measured variations in the plant growth rate and leaf P_n_ and found that plant growth rate and leaf P_n_ also reduced dramatically under waterlogging. However, this response is remarkably suppressed in alfalfa plants with melatonin pretreatment. Such growth and photosynthesis-preserving effects of melatonin have already been shown for numerous plant species under various stress conditions (Mukherjee et al., 2014; Zhao et al., 2017; Zheng et al., 2017).

It has been reported that various environmental abiotic stresses such as temperature stress, salt and water stress, heavy metal stress that stimulated melatonin accumulation in plant species, and this stimulation have been regarded as a self-defense response to external stimuli through the regulation of leaf senescence, antioxidant systems, carbon and nitrogen metabolism in plants (Li et al., 2012; Bajwa et al., 2014; Byeon and Back, 2014; Tan, 2015). In the present study, both waterlogging stress and melatonin pretreatment remarkably increased melatonin levels in alfalfa plants, indicating that waterlogging stress can induce the accumulation of endogenous melatonin in plants as a protective response (Paredes et al., 2009). A greater increase in the melatonin pretreated plants than the untreated ones suggests that melatonin absorbed from outside mainly resulted in melatonin increase in addition to a post-transcriptional regulation of melatonin synthesis (Zheng et al., 2017). The chlorosis is a regular indicator unavoidably happening after severe waterlogging stress, because waterlogging results in over-production of O_2_^⋅-^ and H_2_O_2_, which destroys chlorophyll and leads to the breakdown of chloroplasts (Smethurst and Shabala, 2003). The protective effects of melatonin on Chl degradation and photosynthetic capacity have been investigated previously in other abiotic stresses, such as water deficit and high temperature (Wang et al., 2013; Zhang et al., 2017). The chloroplast has been proved to be the major site for melatonin production, but it was one of the organelles that suffered most from ROS. Plenty of melatonin is required to sustain its structure and function. Therefore, the absorbed and *in vivo*-synthesized melatonin can function together to mitigate waterlogging-induced membrane damage and help alfalfa plants to survive the stress.

Waterlogging decreases photosynthesis in many plant species and develops leaf injury symptoms, such as wilting and chlorosis (Shao et al., 2013), which were also observed in this study. These symptoms developed under waterlogging have been attributed to ethylene production in addition to other restricting factors (Loreti et al., 2016). In our study, ethylene production significantly increased along with the prolonged waterlogging stress treatment in alfalfa, which was inconsistent with previous reports that enhanced ethylene production in perennial pepperweed (*Lepidium latifolium*) (Chen et al., 2002), avocado (*Persea americana*) (Gil et al., 2009), and cotton (*Gossypium hirsutum*) (Najeeb et al., 2018) caused leaf senescence and abscission of leaves and fruits. Ethylene aggravates the effects of abiotic stresses, whereas the detrimental effect of waterlogging on plants can be alleviated by reducing the levels of endogenous ethylene in plants (Najeeb et al., 2015). In this study, alfalfa plants pretreated with melatonin dramatically decreased ethylene production during waterlogging stress, suggesting that the mitigation of melatonin on waterlogging stress in alfalfa are at least partially attributed to reduced ethylene production.

During the ethylene biosynthesis process, both *ACS* and *ACO* are regulated by various external and internal cues to control ethylene production. In addition, the transcription factor ethylene response factor (*ERF*) has also been associated with waterlogging and modulates ethylene response under flooding (Yang et al., 2011). Under waterlogged conditions, more ACC is synthesized in roots and transported to plant shoots, where it is converted to ethylene by *ACO* (Sasidharan and Voesenek, 2015). In the current study, a remarkable upregulation of *ACS* and *ACO* in alfalfa plants under waterlogging stress occurred, along with significantly enhanced ethylene production. Meanwhile pretreatment with melatonin significantly suppressed the *ACS* and *ACO* expression and was accompanied by depressed ethylene production in alfalfa plants under waterlogging. These results suggested that waterlogging stress mitigation by the exogenous melatonin in alfalfa was associated with the ethylene biosynthesis at transcription levels.

Polyamines are polybasic amines that interact with polycationic macromolecules such as DNA, RNA, and proteins (Bouchereau et al., 1999), processes which are positively associated with the endogenous PA levels. In the current study, melatonin pretreatment downregulated ethylene biosynthesis-related gene expression, leading to depressed ethylene production. This process may suppress SAM for ethylene production and moved to PAs biosynthesis. To confirm this hypothesis, free PA content was determined in alfalfa plants after pretreatment with melatonin under waterlogging and it was found that Put, Spd, and Spm contents significantly increased in melatonin pretreated plants. These results indicate that melatonin may strengthen the SAM to PA metabolism, thus increasing waterlogging tolerance in alfalfa plants.

Previous studies indicated that melatonin induces the activity of the PA metabolism enzymes, thus affecting endogenous PA levels under chilling (Zhao et al., 2017). To reveal how melatonin regulates PA levels under waterlogging, the activity of related enzymes and gene expressions related to PA metabolism were determined. The results showed that the gene expression levels and activities of the PA synthesis enzymes, ADC, ODC, SAMDC and also those of the PA catabolism enzymes, DAO, PAO, were dramatically increased under waterlogging, suggesting that PA metabolism was promoted by waterlogging. Furthermore, melatonin pretreatment further increased the activities and gene expression levels of the ADC, ODC and SAMDC enzymes, while it suppressed the activities and gene expression levels of the DAO and PAO enzyme in alfalfa plants under waterlogging. These results indicated that melatonin improved waterlogging tolerance through the regulation of PA metabolism controlled at transcriptional and translational levels in alfalfa plants. It has been well-documented that PAs are involved in the acquisition of plant tolerance to diverse environmental stresses (Groppa and Benavides, 2008; Gupta et al., 2013). *SPMS* and *SPDS* gene expressions are up-regulated by melatonin pretreatment under waterlogging with concomitant suppression of PAO and DAO activity, possibly helping to maintain Spm and Spd content at a high level, which implies that melatonin mitigates waterlogging stress by activating enzymes involved in the PA metabolism to increase the PA levels.

## Conclusion

**FIGURE 11:**
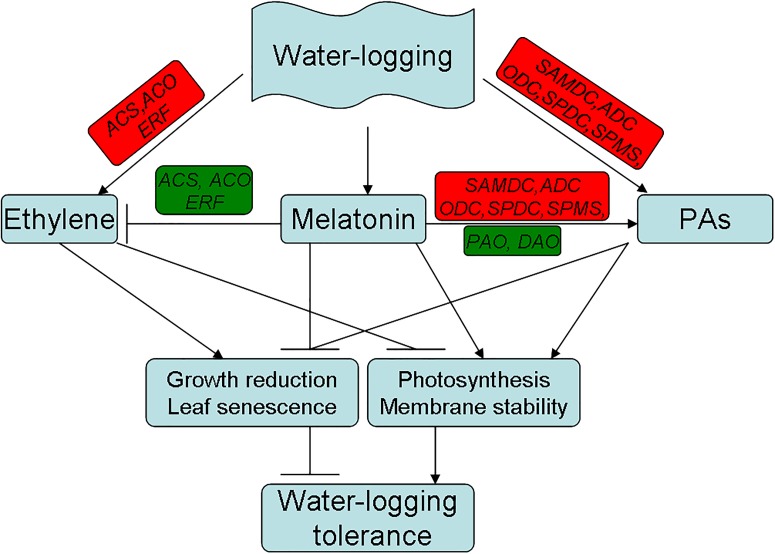
Proposed pathways for the melatonin mediated waterlogging stress response in alfalfa derived from results involving ethylene and PAs metabolism. (Red color indicates induced and green color indicates suppressed for the expression of the gene.)

Based on the above observations, a model for melatonin-mediated waterlogging stress response in alfalfa was proposed in the current study (Figure 11). In this study, we demonstrated that melatonin has significant involvement in mitigating waterlogging stress in alfalfa. The mechanisms through which melatonin alleviates waterlogging injury may be presented as follows. First, melatonin suppressed ethylene production through downregulating the ethylene biosynthesis-related genes and mitigating waterlogging-induced growth reduction, chlorosis and premature senescence in plants. Second, melatonin increases PA content by enhancing the activity and gene expressions of the PA metabolism enzymes. The positive result in this study indicated that melatonin suppressed ethylene production in alfalfa under waterlogging at least partially by reprogramming ethylene and PA biosynthesis. This study provides new evidence that melatonin mitigates waterlogging stress through cross-talk with or directly modulating the metabolic pathways of PAs and ethylene in alfalfa.

## Author Contributions

QZ and XL performed the experiments. NL, DL, and LH conceived, designed, executed, and evaluated of the experiments. ZZ, NL, and LH analyzed the data of the experiments. QZ and LH helped to draft the manuscript. All authors read and approved the final manuscript.

## Conflict of Interest Statement

The authors declare that the research was conducted in the absence of any commercial or financial relationships that could be construed as a potential conflict of interest.

## Abbreviations

ACC: 1-aminocyclopropane-1-carboxylic acid‘CO, ACC oxidase
ACS: ACC synthase
ADC: arginine decarboxylase
DAO: diamine oxidase
MDA: malondialdehyde
ODC: ornithine decarboxylase
PA: polyamine
PAO: polyamine oxidase
Put: putrescine
SAM: *S*-adenosylmethionine
SAMDC: *S*-adenosylmethionine decarboxylase
Spd: spermidine
Spm: spermine
SPDS: spermidine synthase
SPMS: spermine synthase
